# Identification of a novel papillomavirus in oral swabs from giant pandas (*Ailuropoda melanoleuca*)

**DOI:** 10.3389/fvets.2024.1457471

**Published:** 2025-01-03

**Authors:** Yi Zhao, Min Zhao, Wen Zhang

**Affiliations:** Department of Laboratory Medicine, School of Medicine, Jiangsu University, Zhenjiang, Jiangsu, China

**Keywords:** papillomavirus, giant panda, phylogenetic analysis, virus genome, viral metagenomics

## Abstract

To fully characterize papillomavirus diversity in giant pandas (*Ailuropoda melanoleuca*), we identified a novel papillomavirus (named AmPV5, GenBank accession number MZ357114) in oral swabs from giant pandas with the help of viral metagenomics technology in this study. The complete circular genome of AmPV5 is 7,935 bp in length, with a GC content of 39.1%. It encodes five early genes (E1, E2, E4, E6, and E7), two late genes (L1 and L2), and features conserved zinc-binding domains (CXXC- (X)28/29-CXXC) in E6 and E7 genes. E7 protein has an LxCxE domain (pRB binding) in its N-terminal region. The nucleotide sequence of AmPV5 L1 gene shares < 70% identity with other related sequences available in the GenBank database. Phylogenetic analysis indicated that AmPV5 fell within the *Lambdapapillomavirus* genus but formed a monophyletic branch away from other papillomaviruses found in *Ailuropoda melanoleuca, Canis, Felis catus, Panthera uncia, Enhydra lutris*, and *Procyon lotor*. According to the International Committee on Taxonomy of Viruses (ICTV) classification guidelines, AmPV5 is classified as a new species within the *Lambdapapillomavirus* genus. The discovery provides valuable insights into the viral diversity in giant pandas and highlights the need for continued surveillance of wildlife pathogens. Future studies should explore the potential role of AmPV5 in the health and disease ecology of this endangered species.

## 1 Introduction

With the advancement of viral metagenomics, an increasing number of complete genomes of papillomaviruses (PVs) have been identified in a wide range of organisms (1–3). PVs can infect the mucous membranes and squamous epithelium of vertebrates including fish, birds, reptiles, mammals, and even humans, often causing subclinical lesions and benign proliferations (4–6). In severe cases, some of them have potential to transform into malignant tumors, making PVs a continuing concern for both human and animal health even though it is highly specific (7). Various studies have established strong links between PV infections and several types of cancers, such as cervical cancer in humans, squamous cell carcinomas in cats, and papillomas in cows and horses (8–10). Furthermore, it has been shown that PVs have evolved multiple mechanisms to suppress the interferon response and evade host innate immunity effectively, delaying adaptive immune activation and enabling persistent infections that may last months or even years (11, 12). Given the limited genetic diversity of giant pandas and their potential vulnerability to diseases, the role of immune evasion by PVs warrants further investigation to understand its impact on the health and survival of this endangered species.

Papillomaviruses are small, un-enveloped, circular, double-stranded DNA viruses with a genome size of ~8 kb. They contain three functional regions: an early region, a late region, and a non-coding upstream regulatory region (URR) located between L1 and E6 open reading frames (ORFs) (13, 14). The early region encodes six proteins (E1, E2, E4, E5, E6, and E7) involved in viral replication, transcriptional regulation, and cellular transformation (15). The major coat protein L1 and the minor coat protein L2 in the late region play important roles in viral assembly (16). The URR, also known as the locus control region (LCR), contains binding sites for various cellular transcription factors, including E1 and E2 binding sites that regulate transcription and are essential for viral replication (17). The International Committee on Taxonomy of Viruses (ICTV) classifies the *Papillomaviridae* family into 2 subfamilies, over 50 genera, and 130 species based on the highly conserved nucleotide sequence of L1 ORF (13, 18). Members of the same subfamily share < 45% nucleotide sequence identity in their L1 ORF, < 60% for distinct genera, and between 60 and 70% for different species within the same genus (13, 18).

Low rates of reproduction and susceptibility to infectious diseases are significant factors hindering the population growth of giant pandas, an endangered species (19). Although PVs have been detected in giant pandas, virological studies on this species remain limited. To date, only four complete PV genomes (AmPV1 - 4) have been identified in giant pandas, among which AmPV1 and AmPV2 belong to the *Omegapapillomavirus* genus, AmPV4 represents a new species within the *Lambdapapillomavirus* genus, and AmPV3 defines a novel genus, and all their genomes encode the early proteins (E6, E1, and E2) and the late proteins (L2 and L1), typically ranging from 7,000 to 8,000 base pairs (20). Given the importance of this species in global conservation efforts, studying PVs in giant pandas offers a unique opportunity to uncover potential viral threats to their health and contribute to broader wildlife conservation strategies. Understanding PV diversity and its implications in giant pandas could provide insights into disease management and highlight the importance of virological surveillance in endangered species. In our study, we collected nine oral swab samples from captive giant pandas in Chengdu and characterized a novel complete PV in these samples. This discovery enhances our understanding of the PV diversity affecting this endangered species and establishes a foundation for further research into related diseases.

## 2 Materials and methods

### 2.1 Sample collection and preparation

In April 2020, nine oral swab samples from nine different individual giant pandas were collected from giant pandas in Chengdu, Sichuan Province. The collection process involved using disposable sterile gloves, swabs, and tubes. All the samples were transported on dry ice and stored at −80°C. Each swab was placed in a 1.5 mL tube with the head broken off and shaken vigorously for 5 min with 1 mL Dulbecco's Phosphate Buffered Saline (DPBS). All collection tools were sterilized, and the sampling was conducted in a clean environment. To release the virus from the suspension and remove large particles, the samples were incubated for 30 min at 4°C, followed by centrifugation for 10 min at 4°C and 12,000 × g. Finally, the supernatant from each sample was collected into new 1.5 mL tubes.

The sampling procedures were conducted with utmost care to minimize risks to the giant pandas, other associated animals, and the environment. All experiments in this study were approved by the Institutional Animal Care and Use Committee of the Chengdu Research Base of Giant Panda Breeding (Protocol No. 2019013) and the Ethics Committee of Jiangsu University (Protocol Nos. 2015011 and 2017021).

### 2.2 DNA library construction

The shaken and mixed library containing 500 μL sample supernatant was centrifuged at 12,000 × g and 4°C for 5 min before being filtered through a 0.45 μm pore-size filter (Millipore) to remove eukaryotic cells and cell-sized particles (21). Free nucleic acids not protected by viral capsid proteins were degraded using an RNase/DNase digestive enzyme system at 37°C for 60 min (22). This step was performed to degrade both RNA and DNA, targeting host-derived contamination while preserving encapsidated viral nucleic acids. Total nucleic acids (RNA/DNA) were extracted using QIAamp Viral RNA Mini Kit (QIAGEN) following the instructions of the manufacturer. After extraction, the nucleic acids were subjected to reverse transcription using SuperScript III Reverse Transcriptase, and the second strand of cDNA was synthesized with the Klenow enzyme. The DNA libraries were constructed using Nextera XT DNA Sample Preparation Kit (Illumina) to further increase the concentration of viral nucleic acids. Subsequently, the constructed libraries were sequenced on the Illumina NovaSeq 6000 platform, with each sample pool generating 250 bp paired-end reads with double barcodes.

### 2.3 Bioinformatic analysis

The raw data were split and exported based on the Index combination of each library after the downstream machine. Data preprocessing was performed using Geneious Prime and involved debarcoding, trimming, and *de novo* assembling (23). The assembled contigs were analyzed using BLASTx to search for matches against a viral protein database created in our lab. A cut-off E-value of < 10^−5^ was applied for significant hits. Single-stranded reads were also subjected to the same BLASTx analysis (24). These identified sequences were then compared against the in-house non-virus non-redundant (NVNR) protein database to exclude potential false positives. Format conversion was subsequently performed in Megan, where sequence classification information was presented visually (25). Our previous study found that before the giant panda (*Ailuropoda melanoleuca*) had mild diarrhea in Chengdu Research Base of Giant Panda Breeding (April, 2020), *Papillomaviridae* were observed in the highest abundance of all the detected viral reads at the family level (26). Here, to fully characterize papillomavirus diversity, contigs belonging to the *Papillomaviridae* family were selected and assembled using Geneious Prime. Open reading frames (ORFs) and genome annotations were also predicted in Geneious Prime based on the comparisons to the Conserved Structural Domain Database (CDD) (27). SWISS-MODEL was used to predict the tertiary structure of viral proteins (28, 29). Finally, a complete papillomavirus genome sequence with low nucleotide similarity to other papillomaviruses was obtained and included in subsequent analyses.

### 2.4 Phylogenetic analysis

The amino acid sequence of the L1 protein predicted in our study was compared with related protein sequences, including those of the closest viral relatives identified through BLASTp searches in GenBank, as well as the reference sequences from different genera and species of *Papillomaviridae*. All these sequences were downloaded and aligned using MUSCLE with the E-INS-I algorithm in MEGA (30, 31). A phylogenetic tree was constructed using the “prset aamodelpr=mixed” model in MrBayes, employing the Markov chain Monte Carlo (MCMC) method (32). Additionally, a Maximum Likelihood tree was constructed to validate the Bayesian inference tree using MEGA (33).

### 2.5 Quality control

To prevent cross-contamination of samples and nucleic acid degradation, all experimental procedures were strictly conducted in accordance with the relevant guidelines. All materials in direct contact with nucleic acid samples were sterile and free of enzymes. Nucleic acid samples were dissolved in DEPC-treated water to maintain integrity, and all extraction and processing steps were performed under sterile conditions using a biological safety cabinet to ensure sample purity.

## 3 Results

### 3.1 Overview of the virome

**Figure 1:**
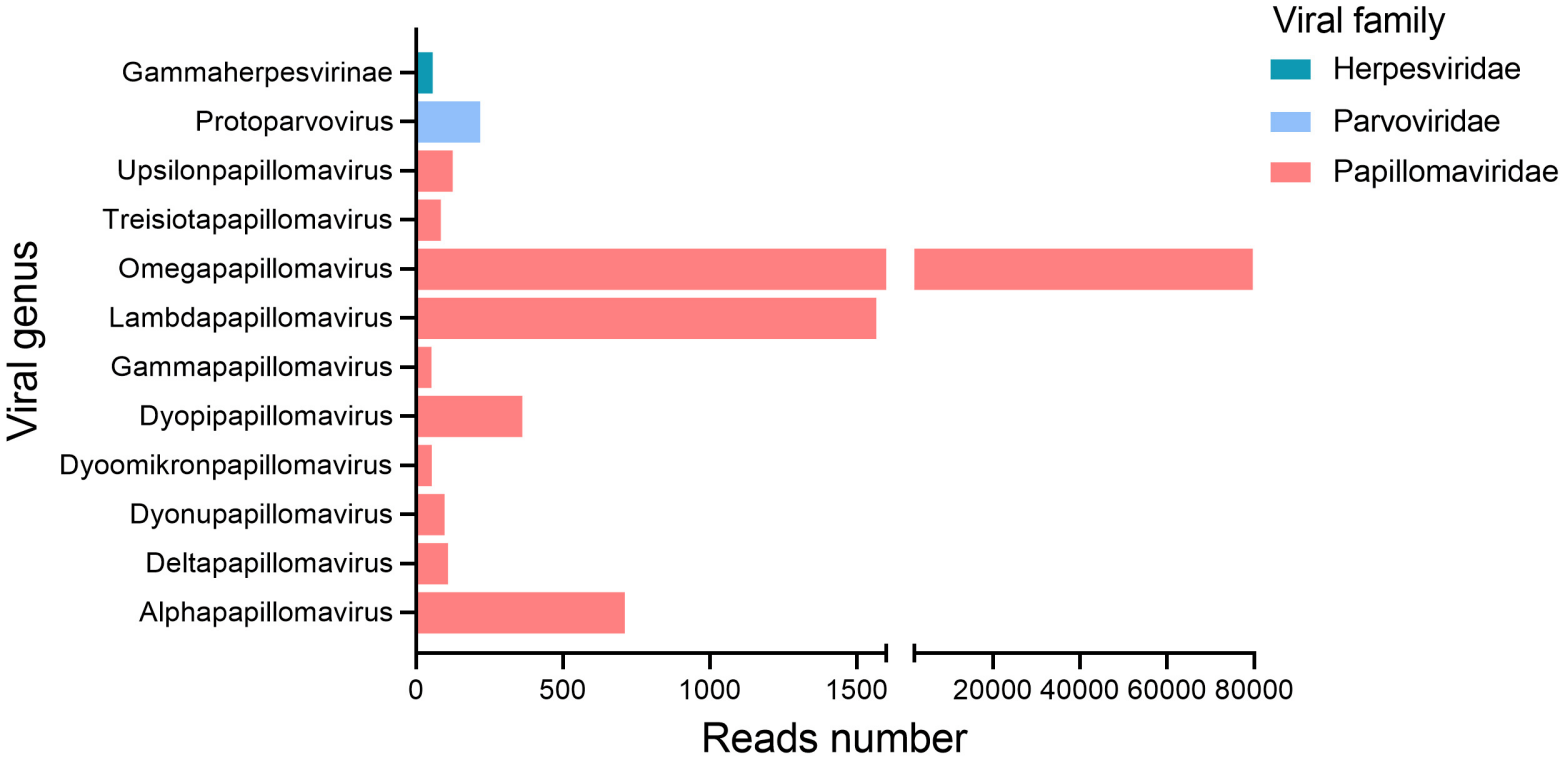
Bar plots showing taxonomic category and viral abundance in the gporal003 library generated from nine giant panda oral swab samples.

All nine oral swab samples from giant pandas were pooled into a library (gporal003) for next-generation sequencing (NGS), generating a total of 1,063,578 raw sequence reads, among which 8.73% (*n* = 92,889) reads showed similarity to known eukaryotic viruses. The remaining 91.27% (*n* = 970,689) of the sequencing data aligned with sequences from eukaryotes, prokaryotes, bacteriophages, or sequences with no significant similarity to any amino acid sequences in the NR database. At the family level, almost all eukaryotic viral reads (*n* = 92,654) were assigned to three families of *Papillomaviridae* (*n* = 92,185), Parvoviridae (*n* = 240), Herpesviridae (*n* = 229) (Figure 1). Reads related to *Papillomaviridae* were overwhelmingly dominant. At the genus level, these reads were classified into 10 distinct papillomavirus genera, including *Alphapapillomavirus, Deltapapillomavirus, Dyonupapillomavirus, Dyoomikronpapillomavirus, Dyopipapillomavirus, Gammapapillomavirus, Lambdapapillomavirus, Omegapapillomavirus, Treisiotapapillomavirus*, and *Upsilonpapillomavirus*.

### 3.2 Genomic characteristics

**Figure 2:**
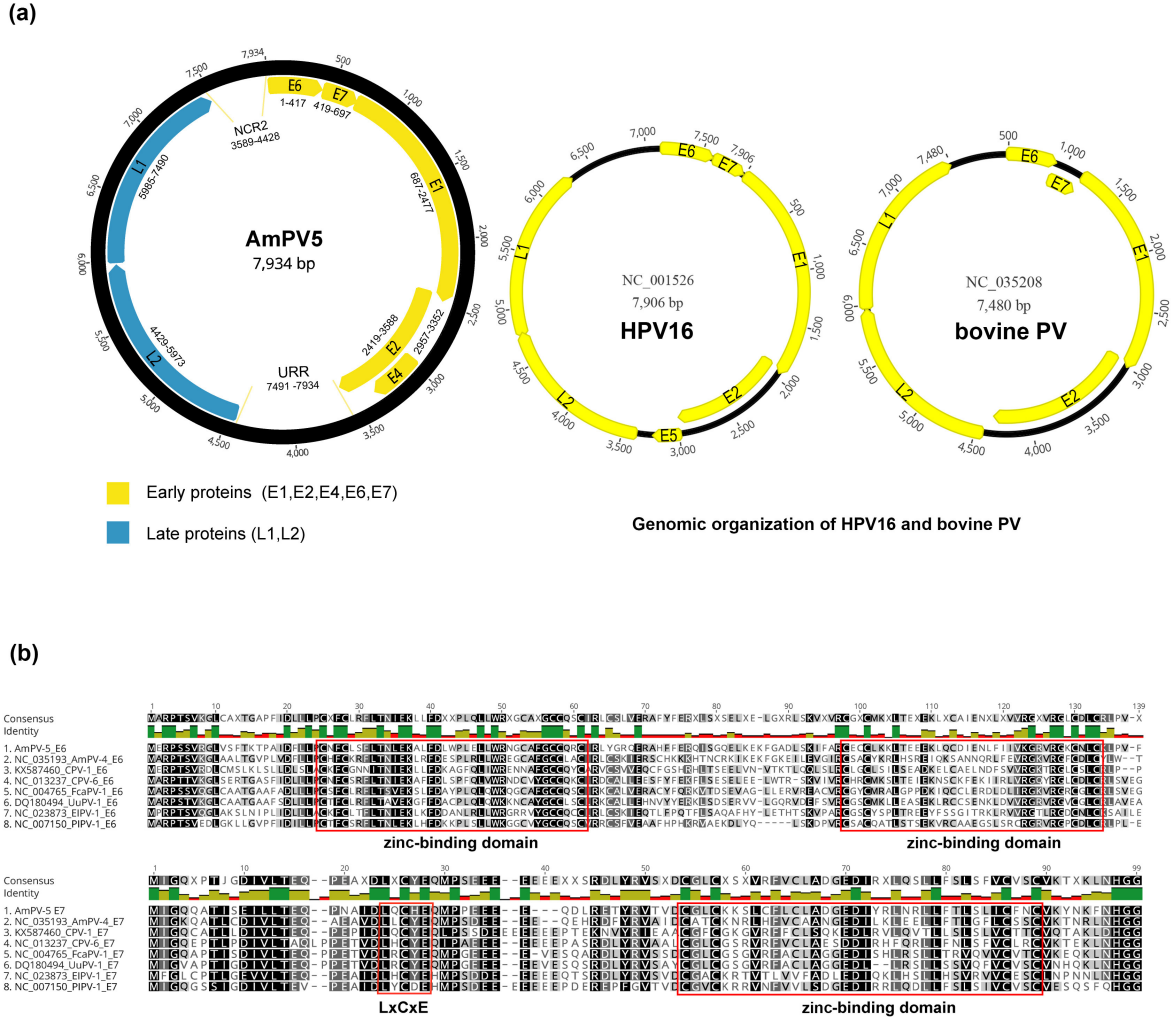
**(A)** Schematic presentation of the genome organization of AmPV5. Genomic structure diagram of HPV16 and bovine PV. **(B)** Alignment of amino acid of E6 and E7 proteins between AmPV5 and several related PVs.

The circular genome of AmPV5 is 7,935 bp in length with a GC content of 39.1%. It contains three main regions: an early region encoding five proteins (E1, E2, E4, E6, and E7), a late region encoding two proteins (L1 and L2), and a non-coding URR region located between the E2 and L2 ORFs. Their lengths are as follows: E1 is 1,791 bp, E2 is 1,170 bp, E4 is 396 bp, E6 is 417 bp, E7 is 279 bp, L1 is 1,506 bp, and L2 is 1,545 bp. Additionally, there is a small non-coding region named NCR2 positioned between the L1 and E6 ORFs at positions from 3,589 to 4,428bp (Figure 2A). Non-coding regions like the URR and NCR2 play key roles in transcription regulation of papillomaviruses. These regions often contain promoter and enhancer elements that influence viral gene expression. They may also mediate interactions with host factors, potentially affecting viral replication and pathogenicity. Future functional studies could provide more insight into their specific roles in AmPV5. The first nucleotide was defined as the initiation codon of the E6 gene in AmPV5.

Analysis of the nucleotide and amino acid sequences revealed several classical signature sites in AmPV5 compared to other PVs. E1 and E2 genes of PVs play essential roles in viral DNA replication and gene regulation. An ATP-binding site of the ATP-dependent DNA helicase (GPPNTGKS) is found in AmPV5 E1 at position 424–431. The presence of this motif led to the speculation that this protein might exhibit ATPase activity and helicase activity similar to those of the simian virus 40 (SV40) and polyomavirus T antigens (34). The E2 gene consists of a N-terminal transactivation domain and a C-terminal DNA binding domain. The N-terminal domain is essential for regulating viral gene transcription, and influence the expression of early genes such as E6 and E7 (35). The C-terminal domain is responsible for the dimerization of E2 proteins (36). Recent studies have highlighted the critical interactions between E1 and E2, and their combined functions are crucial for maintaining the viral genome in a stable episomal form and ensuring efficient replication. There are some differences in the structure of the AmPV5 genome compared to other papillomaviruses. For instance, AmPV5 lacks the E5 gene, which in high-risk PVs like Human papillomavirus type 16 (HPV16) is associated with immune modulation and oncogenesis (37). However, compared with HPV16 and bovine PV, AmPV5 shows a longer URR area, which is located between the L1 and E6 ORFs and contains the viral origin of replication as well as binding sites for viral and cellular transcription factors. E6 ORF features four CXXC motifs, forming two conserved zinc-binding domains: CXXC-X29-CXXC and CXXC-X28-CXXC, separated by 36 amino acids (Figure 2B). A similar zinc-binding domain (CXXC-X28-CXXC) is located at the C-terminus of E7 ORF, which plays a key role in the formation of E7 dimer (38, 39). Furthermore, the E7 ORF contains a conserved motif (LXCXE), known as the core binding region for E7 and the MCV (Merkel cell polyomavirus). Large T oncoprotein targeting the retinoblastoma tumor suppressor protein (pRB) (40, 41). These results provide valuable insights for future studies on the interactions of AmPV5 with its host.

### 3.3 Projections of conservative structure

**Figure 3:**
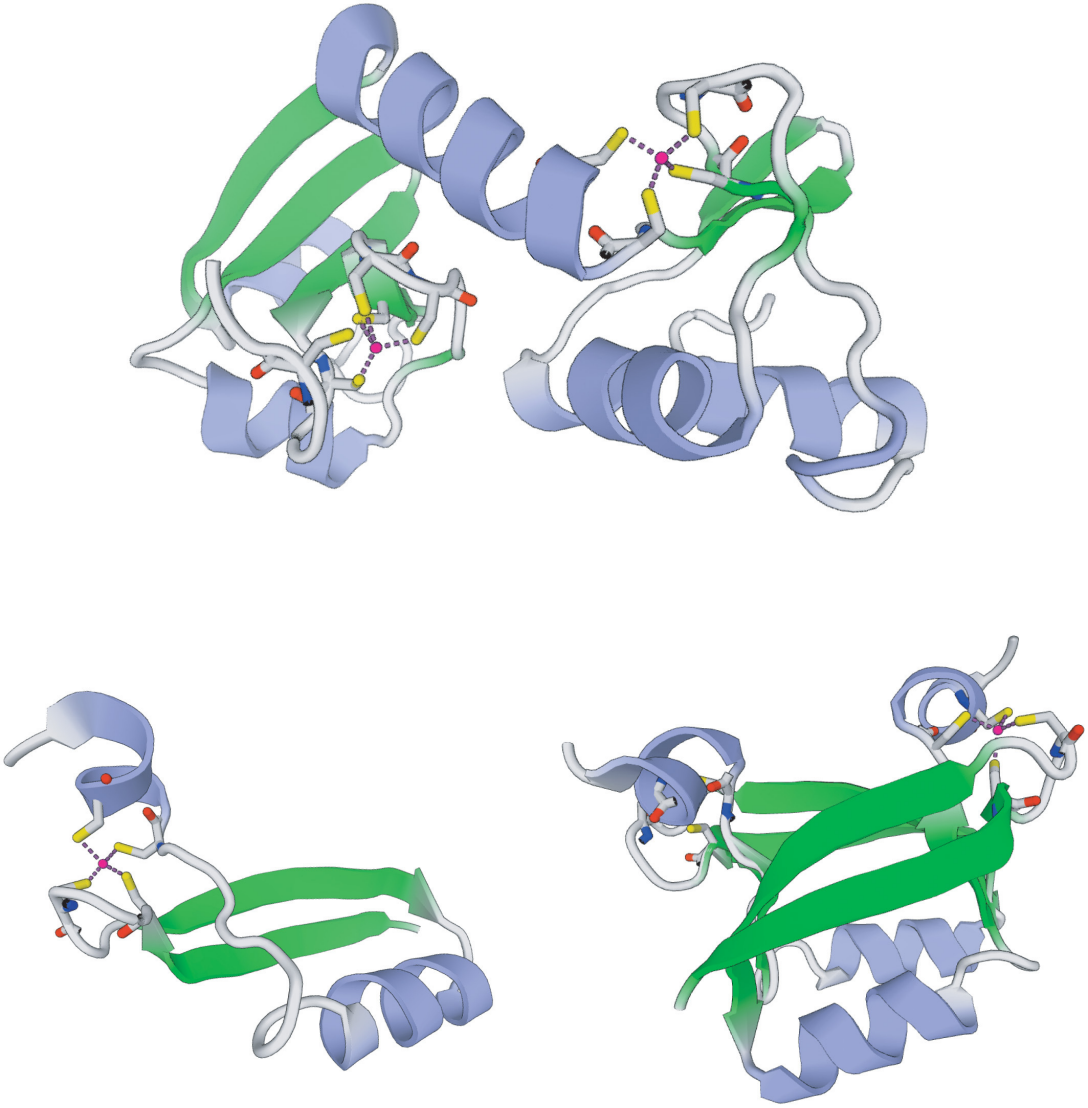
Projections of the tertiary structural of AmPV5 E6 and E7 proteins.

Given that the transformation of host cancer cells by HPV is primarily mediated by E6 and E7 genes, we conducted tertiary structure predictions of the characteristic conserved regions (CXXC and LXCXE) on AmPV5 E6 and E7 genes (Figure 3) (6, 41). Our analysis revealed that E6 protein of AmPV5 contains four CXXC conserved regions, forming two stable zinc-binding domains, whereas E7 protein has two CXXC regions, forming a single strong zinc-binding domain. Remarkably, this domain drives the formation of a unique dimeric structure, similar to what has been observed in high-risk genital HPVs involved in the inactivation of human cancer-related proteins such as histone deacetylase (42). The CXXC motif may enhance protein stability through zinc binding, while the LXCXE motif facilitates interactions with the retinoblastoma protein (pRB), disrupting cell cycle control. These features suggest AmPV5 may have enhanced host adaptation and potential oncogenic properties. Functional validation is an important next step to confirm these effects.

### 3.4 Identification of the novel species of *Lambdapapillomavirus* and phylogenetic analysis

The L1 gene of AmPV5 showed 69.34% nucleotide sequence identity to that of the best-matched papillomavirus strain ElkPV-2 (GenBank accession number OQ746290) in BLASTx searching. Based on the ICTV classification criteria for PVs, AmPV5 represents a new species. A previous study has identified four complete PVs in giant pandas (AmPV1, AmPV2, AmPV3, and AmPV4) (20). Therefore, the new papillomavirus identified in this study was named as AmPV5. Multiple sequence alignment revealed that AmPV5 shared < 70% identity in both nucleotide and amino acid sequences of L1 gene with other PVs discovered in giant pandas (Tables 1, 2). Among them, AmPV5 and AmPV4 (GenBank accession number NC_035193) shared the highest nucleotide sequence identity of 68.79% in L1 genes and belonged to the same genus, *Lambdapapillomavirus*. Conversely, AmPV5 and the other three giant panda PVs exhibited < 60% nucleotide identity, placing them in different viral genera. Alignment of the L1 protein amino acid sequences from these five giant panda-origin PV strains confirmed these findings.

**Table 1:** Nucleotide sequence alignment of the L1 genes of all AmPVs.

**Nucleotide alignment (% identity)**	**AmPV5-L1**	**AmPV1-L1**	**AmPV2-L1**	**AmPV3-L1**	**AmPV4-L1**
AmPV5-L1		56.35%	55.49%	52.36%	68.79%
AmPV1-L1	56.35%		68.74%	57.34%	58.33%
AmPV2-L1	55.49%	68.74%		58.10%	55.95%
AmPV3-L1	52.36%	57.34%	58.10%		54.02%
AmPV4-L1	68.79%	58.33%	55.95%	54.02%	

**Table 2:** L1 Amino acid sequence alignment of the L1 proteins of all AmPVs.

**Protein alignment (% identity)**	**AmPV5-L1**	**AmPV1-L1**	**AmPV2-L1**	**AmPV3-L1**	**AmPV4-L1**
AmPV5-L1		49.12%	50.68%	51.39%	66.80%
AmPV1-L1	49.12%		73.37%	58.30%	51.28%
AmPV2-L1	50.68%	73.37%		57.00%	53.15%
AmPV3-L1	51.39%	58.30%	57.00%		53.09%
AmPV4-L1	66.80%	51.28%	53.15%	53.09%	

**Figure 4:**
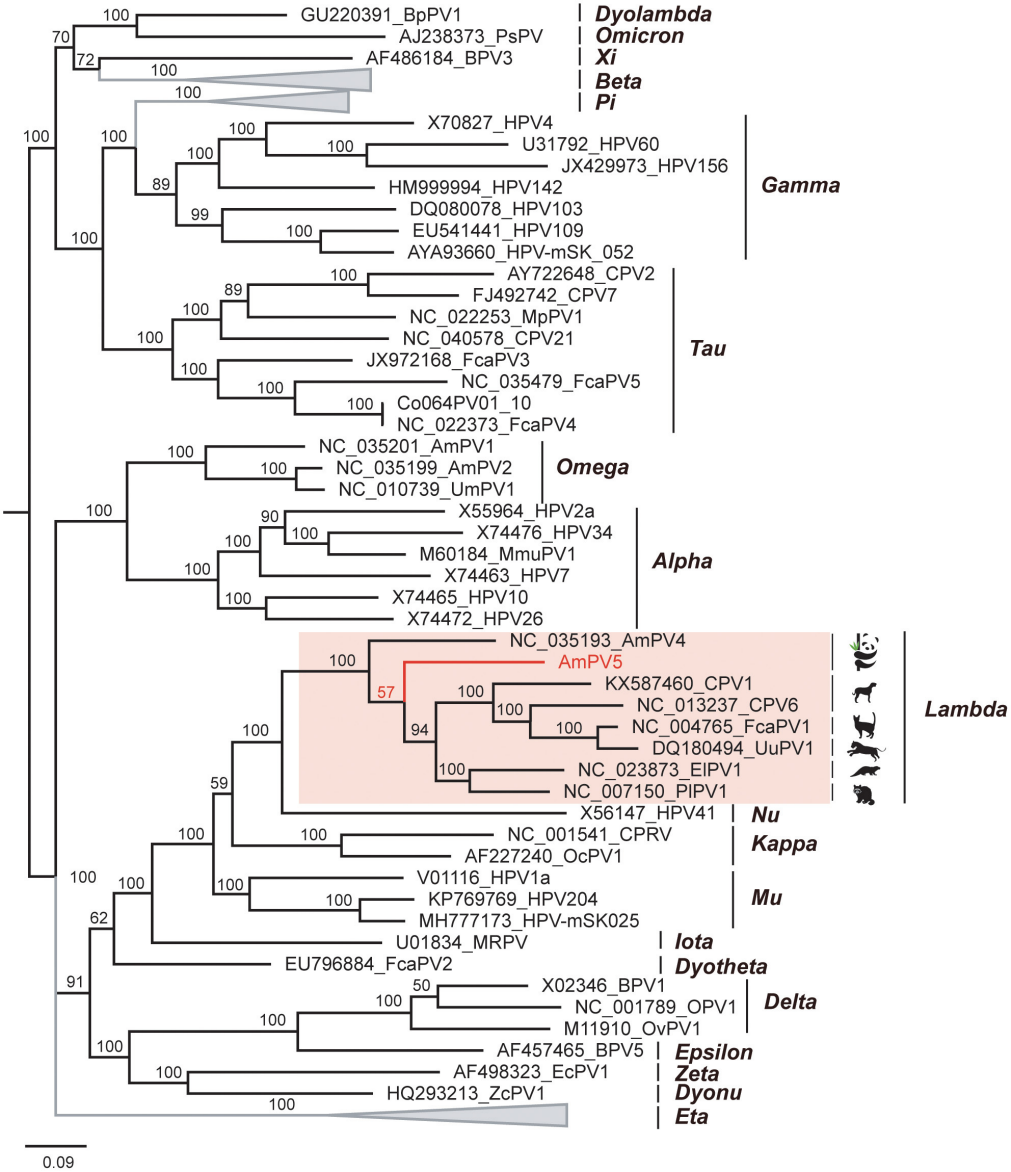
Phylogenetic analysis of PVs. The newly discovered papillomavirus, AmPV5 is highlighted in red.

To elucidate the genetic relationship of this novel papillomavirus with others in mammals, we conducted amino acid sequence alignment of L1 proteins and constructed a Bayesian inference tree (Figure 4). The alignment involved 62 sequences, including AmPV5, top matches identified through BLASTp searches of L1 protein, and representative papillomavirus reference sequences from various genera and species in GenBank. Results revealed that AmPV5 shares the highest amino acid sequence similarity (70.38%) with the L1 protein of a papillomavirus isolated from *Enhydra lutris* (GenBank accession number NC_023873). Phylogenetically, AmPV5 were grouped together with other 6PVs from *Ailuropoda melanoleuca, Canis, Felis catus, Panthera uncia, Enhydra lutris, and Procyon lotor* within the *Lambdapapillomavirus* genus, amino acid similarities of 60–70%. The Maximum-likelihood tree was constructed using MEGA supported these above findings, confirming that AmPV5 is indeed a new species of *Lambdapapillomavirus* genus.

## 4 Discussion

Many viruses have evolved to establish a homeostatic equilibrium with their hosts over extended periods, often resulting in persistent asymptomatic infections (43). However, the spread and mutation of viruses between species can significantly alter their pathogenicity and virulence, particularly in immunocompromised hosts who may develop health complications post-infection (44, 45). Our findings indicate that AmPV5 infects giant pandas without causing concurrent disease. Conversely, Zhang et al., reported that the number of *Papillomaviridae* reads and the papillomavirus positivity rate in healthy pandas were much lower compared to those in the deceased giant pandas, suggesting a potential causative role of papillomaviruses in fatal cases (20). Furthermore, instances of papillomavirus pathogenicity in humans and other species are well-documented, and cross-species transmission events have been reported (44, 46). Therefore, there is an urgent need to employ viral metagenomics approaches to explore and characterize potential pathogenic viruses in humans, animals, and the environment, expanding our understanding of candidate pathogenic agents.

In this study, we successfully assembled and annotated a complete *Lambdapapillomavirus* in oral swabs from giant pandas, named AmPV5 MZ357114. The genome of AmPV5 is 7,935 bp in length with a GC content of 39.1%, encoding both the early region (E1, E2, E4, E6, E7) and the late region (L1 and L2). A BLASTx search in GenBank based on the conserved L1 sequence revealed a 69.34% identity with the closest match (ElkPV2, GenBank accession number OQ746290), while the BLASTn search showed the highest amino acid similarity (70.38%) with a papillomavirus from *Enhydra lutris* (ElPV1, GenBank accession number NC_023873). A Bayesian inference tree was constructed based on the amino acid sequences of the L1 ORF, and the viruses from *Enhydra lutris, Ailuropoda melanoleuca, Canis, Felis catus, Panthera uncia*, and *Procyon lotor* were clustered in a branch within *Lambdapapillomavirus* genus, suggesting potential interspecies transmission. Additionally, AmPV5 was classified as a new species within the genus *Lambdapapillomavirus* according to ICTV classification guidelines. Our analysis identified key regions on AmPV5 E6 and E7 proteins: CXXC and LXCXE motifs. These conserved structures are crucial in papillomavirus interactions with host physiology and biochemistry, contributing to disease induction (47). Specifically, E6 has been implicated in p53 oncogene degradation by inhibiting CBP/p300 co-activator transcription, preventing apoptosis (48). E7 affects cellular transformation through conserved LXCXE motifs and critical zinc-binding regions, contributing to cervical cancer pathogenesis (49, 50). In our study, we find that E5 is missing in AmPV5. Actually, many but not all papillomaviruses encode E5 proteins, which are short, membrane-associated proteins with transforming activity. In non-mammalian PVs or certain low-risk mammalian PVs, the E5 gene is frequently absent, suggesting that its functions may not be essential in all host-virus interactions (51). Notably, all currently identified PVs infecting giant pandas lack the E5 gene, which may represent a characteristic feature of PVs specific to this host species. This consistent absence may reflect evolutionary adaptations to the host environment or particular aspects of the viral life cycle in giant pandas. Further investigation into these key proteins and their binding regions in new papillomavirus species is essential to elucidate papillomavirus-host interactions.

In addition to papillomaviruses (PVs), several other viruses have been identified in giant pandas, some of which may pose potential risks to other species, including humans. For instance, canine distemper virus (CDV) has caused outbreaks in giant panda populations, raising concerns about its potential for cross-species transmission (52). Similarly, feline panleukopenia parvovirus (FPV), known to infect a range of carnivorous mammals, and certain enteric viruses, such as rotaviruses and coronaviruses, have been detected in giant pandas (53). While there are no documented cases of direct virus transmission from giant pandas to humans, the presence of zoonotic pathogens highlights the importance of biosecurity in conservation settings (54). These findings underscore the need for ongoing surveillance of viral infections in giant pandas to protect both the species and public health.

Overall, this study significantly expands our understanding of papillomavirus diversity in giant pandas, enriching the viral ontogenetic database related to these animals and their surroundings. Although no direct evidence was found linking AmPV5 to disease in giant pandas, the structural features of E6 and E7 suggest the virus may affect the host cell cycle or immune response, highlighting the need for further research. Future studies could include longitudinal studies to monitor the prevalence and transmission dynamics of AmPV5 across different panda populations, regions, and age groups, thereby assessing potential health risks. Additionally, functional validation of E6 and E7 proteins through cell culture or animal models could help determine their impact on host cellular pathways and pathogenic potential. These efforts will provide a comprehensive understanding of AmPV5′s role in panda health and conservation, supporting proactive measures against emerging diseases.

## Data Availability

The datasets presented in this study can be found in online repositories. The names of the repository/repositories and accession number (s) can be found in the article/supplementary material.
